# Association of Hospital-Level Intensive Care Unit Use and Outcomes in Older Patients With Isolated Rib Fractures

**DOI:** 10.1001/jamanetworkopen.2020.26500

**Published:** 2020-11-19

**Authors:** Jessica A. Bowman, Miriam Nuño, Gregory J. Jurkovich, Garth H. Utter

**Affiliations:** 1 Department of Surgery, University of California, Davis; 2 Department of Public Health Sciences, University of California, Davis; 3 Department of Surgery Outcomes Research Group, University of California, Davis; 4 Division of Trauma and Acute Care Surgery, Department of Surgery, University of California, Davis

## Abstract

**Importance:**

The optimal level of care for older patients with rib fractures as an isolated injury is unknown.

**Objectives:**

To characterize interhospital variability in intensive care unit (ICU) vs non-ICU admission of older patients with isolated rib fractures and to evaluate whether greater hospital-level use of ICU admission is associated with improved outcomes.

**Design, Setting, and Participants:**

This cohort study included trauma patients aged 65 years and older with isolated rib fractures who were admitted to US trauma centers participating in the National Trauma Data Bank between January 1, 2015, and December 31, 2016. Patients were excluded if they had other significant injuries, were intubated or had assisted respirations in the emergency department (ED), or had a Glasgow Coma Scale (GCS) score of less than 9 in the ED. Hospitals with fewer than 10 eligible patients were excluded. Data analysis was conducted from May 2019 through September 2020.

**Exposures:**

Admission to the ICU.

**Main Outcomes and Measures:**

Composite of unplanned intubation, pneumonia, or death during hospitalization.

**Results:**

Among 23 951 patients (11 066 [46.2%] women; mean [SD] age, 77.0 [7.2] years) at 573 hospitals, the median (interquartile range) proportion of ICU use was 16.7% (7.4%-32.0%), but this varied from a low of 0% to a high of 91.9%. The composite outcome occurred in 787 patients (3.3%), with unplanned intubation in 317 (1.3%), pneumonia in 180 (0.8%), and death in 451 (1.9%). Accounting for the hierarchical nature of the data and adjusting for propensity scores reflecting factors associated with ICU admission, receiving care at a hospital with the greatest ICU use (quartile 4), compared with a hospital with the lowest ICU use, was associated with decreased likelihood of the composite outcome (adjusted odds ratio, 0.71; 95% CI, 0.55-0.92).

**Conclusions and Relevance:**

In this study, admission location of older patients with isolated rib fractures was variable across hospitals, but hospitalization at a center with greater ICU use was associated with improved outcomes. It may be warranted for hospitals with low ICU use to admit more such patients to an ICU.

## Key Points

**Question:**

Is a greater hospital-level tendency to admit older patients with rib fractures as an isolated injury to the intensive care unit (ICU) associated with improved outcomes for those patients?

**Findings:**

In this cohort study of 23 951 patients aged 65 years and older, ICU use per trauma center varied from 0% to 92% of patients with isolated rib fractures. Greater ICU use was associated with a reduction in a composite adverse outcome of death, unplanned intubation, or pneumonia.

**Meaning:**

These findings suggest that admission to an ICU may improve the outcomes of older patients with isolated chest wall injuries.

## Teaser

This cohort study characterizes interhospital variability in intensive care unit vs non–intensive care unit admission of older patients with isolated rib fractures and evaluates whether greater hospital-level use of intensive care units is associated with improved outcomes.

## Introduction

Despite typically sustaining lower-energy mechanisms of injury, older trauma patients (ie, ≥65 years) with chest wall injuries consistently have worse outcomes, including higher risk of pneumonia and death, compared with younger patients.^1,2,3,4^ Accordingly, physicians tend to treat these patients aggressively, focusing on pulmonary hygiene and pain management and sometimes admitting patients to an intensive care unit (ICU) with the goal of preventing complications.

Recent guidelines from the Western Trauma Association (WTA), based on observational studies and institutional experience, recommend that older patients with at least 2 significant rib fractures be admitted to an ICU for close monitoring for at least 24 hours, following which those without respiratory decline may safely be transferred from the ICU.^5^ The authors acknowledge that patients with good baseline function, pain control, and inspiratory effort may not need ICU monitoring. While the putative goals of ICU admission are to optimize nursing attention, medication dosing, and access to respiratory therapists, ICU admission may cause unintended disturbances in the diurnal cycle, increased sedative and opiate administration, increased rates of delirium, and unnecessary interventions, all of which may contribute to poor outcomes.^6,7^ Additionally, ICU care is a limited resource that must be appropriately shared among other critically ill or injured patients.

One recent study identified only 42% adherence to this WTA recommendation across all trauma centers participating in the National Trauma Data Bank (NTDB) and found that adherence to this practice was associated with a modest improvement in mortality.^8^ However, this study did not exclude patients with significant nonthoracic injuries, characterize ICU admission practices at the level of centers, or consider outcomes other than mortality. Additional evidence that ICU care improves outcomes in this population would be helpful to justify widespread implementation of this practice.

We sought to (1) describe national variability in admission disposition (ICU vs non-ICU) of older patients with isolated rib fractures and (2) determine whether hospital-level ICU use is associated with improved inpatient outcomes for this population. We hypothesized that greater hospital-level ICU use would be associated with improved outcomes.

## Methods

### Study Design

We performed a retrospective patient-level cohort study using the 2015 and 2016 NTDB, which contains submissions from more than 700 hospitals nationwide, including most level I and II trauma centers. The study was approved by the University of California, Davis, institutional review board, which waived the requirement for informed consent because it was not possible to contact participants and the study presented minimal risk. This study followed Strengthening the Reporting of Observational Studies in Epidemiology (STROBE) reporting guideline.

### Patient Identification

We included older patients (ie, ≥65 years) with traumatic rib fracture(s), defined by an *International Classification of Diseases, Ninth Revision, Clinical Modification* (*ICD-9-CM*); *International Classification of Diseases, 10th Revision, Clinical Modification* (*ICD-10-CM*); *International Classification of Diseases, 10th Revision, Canada*; or Abbreviated Injury Scale (AIS) code (eAppendix 1 in the Supplement) who were admitted to a center participating in the NTDB between January 1, 2015, and December 31, 2016. We used both *ICD-9-CM* and *ICD-10-CM* codes because *ICD-10-CM* was implemented on October 1, 2015. Our focus was specifically on the association of rib fractures with the decision of where to admit the patient, so we excluded patients with a significant injury in any region other than the thorax (nonthoracic AIS >1) or significant thoracic injury not related to the chest wall (thoracic AIS >2, related to vascular, cardiac, bronchial, or esophageal injury or injuries resulting in shock [>20% blood loss]) (eTable 1 and eTable 2 in the Supplement). We excluded patients who had another potential indication for ICU admission, such as those who were intubated or had assisted respiration in the emergency department (ED) and those with Glasgow Coma Scale (GCS) score of less than 9 in the ED. We excluded patients with an ED disposition other than the ward, telemetry or stepdown unit, or ICU (eg, patients admitted to an observation unit as their ED disposition) because it was not possible to determine what level of care they subsequently received.

We defined exposure status as the hospital-level proportion of eligible patients admitted to an ICU from the ED (ie, ICU use) during the study period. We ranked hospitals in quartiles based on ICU use. Because the NTDB combines the concepts of intermediate care units and telemetry monitoring in a telemetry or stepdown unit category of ED disposition, we considered both floor beds and telemetry or stepdown unit as non-ICU admissions. We excluded hospitals that admitted fewer than 10 eligible patients during the study period to ensure robustness of the exposure status.

### Patient and Hospital Characteristics

Patient-level characteristics included age, sex, comorbid conditions, physiologic parameters (eg, initial ED vital signs and GCS score), and injury characteristics (eg, mechanism of injury, number of rib fractures [categorized by combining information from both AIS predot codes and *ICD* codes], pulmonary contusion, hemothorax or pneumothorax, AIS scores, and Injury Severity Score [ISS]). Hospital-level characteristics included trauma center level, university affiliation, hospital size, number of ICU beds, number of trauma attendings, geographical region, presence of a telemetry or stepdown unit, proportion of all trauma patients in the NTDB aged 65 years or older, and proportion of all trauma patients injured by blunt mechanism.

### Outcomes

The primary outcome was a composite adverse outcome of pneumonia, unplanned intubation, or death during the index hospitalization. Within the NTDB, unplanned intubation is defined as placement of an endotracheal tube and mechanical ventilation for respiratory or cardiac failure. Prespecified secondary outcomes included the individual components of the composite outcome (ie, pneumonia, unplanned intubation, and death), adult respiratory distress syndrome, unplanned ICU transfer, total ICU length of stay, and total ventilator days. The NTDB defines unplanned ICU transfer to include both transfer to an ICU after initial admission to a floor bed and return to an ICU after transfer out; admission to an ICU after a planned surgical procedure is not included. We evaluated unplanned ICU transfer as a secondary outcome to capture circumstances in which patients required an increased level of care during their hospital course but may not have developed pneumonia or required intubation. We assumed missing total ICU length of stay or ventilator days corresponded to no ICU or ventilator use.

### Statistical Analysis

We evaluated the agreement in hospital-level quartile of ICU use between 2015 and 2016 with Cohen κ coefficient, using weights of 1 − [(*i* − *j*)/(*k* − 1)]^2^, with *i *and *j *indicating the row and column of ratings (eg, quartile in 2015 vs quartile in 2016) and *k *indicating the number of possible ratings (ie, 4). We performed descriptive analyses to compare patient-level and hospital-level characteristics across the quartiles of ICU use.

As a strategy to address confounding, we evaluated the validity of instrumental variable methods that would treat hospital-level ICU use as a preference instrument (eAppendix 2, eTable 4, and eTable 5 in the Supplement). The concept is that a construct of hospital-level ICU use would be correlated with actual ICU use and associated with outcomes only through its association with ICU use, plausibly functioning effectively as a randomly assigned intervention. However, our analyses identified that hospital-level ICU use failed to satisfy the exogeneity assumption of an instrument, thus invalidating its consideration as an instrumental variable.

Therefore, we used generalized linear mixed models with a logit link, which account for clustering of observations within hospitals and allow modeling of hospital-specific random effects, to evaluate the association between hospital-level quartile of ICU use and binary outcomes (composite outcome, pneumonia, unplanned intubation, death, adult respiratory distress syndrome, and unplanned ICU transfer). We adjusted for all potential confounders at the patient level and hospital level (eAppendix 2 and eTable 6 in the Supplement) with summary propensity scores rather than individual variables. We derived propensity scores from probabilities of exposure (ICU admission vs non-ICU admission) estimated via a logistic regression model and incorporated the scores into the mixed-level models as a covariate indicating the probability of ICU admission (ranging from 0 to 1). We used a similar approach with a Poisson link to evaluate the association between the quartile of ICU use and both total ICU length of stay and ventilator days, reported as incidence rate ratios.

We performed sensitivity analyses to examine whether alternative definitions of the study population, exposure status, and outcomes might influence any observed associations. These included: (1) expanding the definition of ICU admission to include patients admitted to a telemetry or stepdown unit; (2) expanding the study cohort to include patients with nonthoracic injuries with an AIS score of 2 or less; (3) expanding the cohort to include patients admitted to observation status (considered non-ICU); (4) restricting the cohort to patients with at least 2 fractured ribs; (5) restricting the cohort to patients with at least 3 fractured ribs; (6) restricting the cohort to hospitals with at least 20 eligible patients during the study period; and (7) expanding the definition of the composite outcome to include discharge to hospice.

We used Stata version 14 (StataCorp) and SAS version 9.4 (SAS Institute) for statistical analyses. We conducted 2-sided tests and defined statistical significance as *P* < .05, without correction for multiple testing. We analyzed data from May 2019 through September 2020.

## Results

Among 1 886 530 records in the NTDB during 2015 and 2016, 23 951 hospitalizations at 573 hospitals met inclusion criteria (Figure) (11 066 [46.2%] women; mean [SD] age 77.0 [7.2] years) (Table 1). The proportion of patients per hospital admitted to an ICU ranged from 0% to 91.9% (quartile 1: 0%-7.3%; quartile 2: 7.4%-16.6%; quartile 3: 16.7%-32.0%; quartile 4: 32.1%-91.9%) (eFigure in the Supplement). ICU admission from the ED occurred for 5923 patients (24.7% overall; quartile 1: 3.8%; quartile 2: 11.2%; quartile 3: 24.3%; quartile 4: 50.0%; *P* < .001). Cohen κ demonstrated substantial agreement in quartile of ICU use between years 2015 and 2016 (κ = 0.65).

**Figure.**
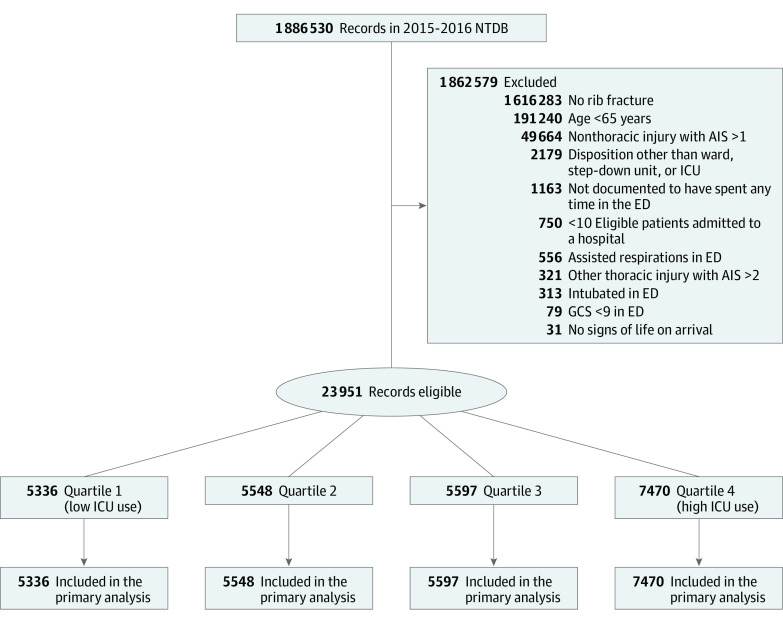
Derivation of the Study Cohort AIS indicates Abbreviated Injury Scale; ED, emergency department; GCS, Glasgow Coma Scale; ICU, intensive care unit; and NTDB, National Trauma Data Bank.

**Table 1.** Baseline Characteristics of the 23 951 Older Patients With Isolated Rib Fractures, by Quartile of Hospital-Level ICU Use

Patient characteristic	Hospital-level proportion of older patients with isolated rib fractures admitted to an ICU, No. (%)				*P* value	Standardized difference between quartiles 1 and 4
	Quartile 1, 0%-7.3% (n = 5336)	Quartile 2, 7.4%-16.6% (n = 5548)	Quartile 3, 16.7%-32.0% (n = 5597)	Quartile 4, 32.1%-91.9% (n = 7470)		
Age, mean (SD), y	77.4 (7.3)	77.1 (7.2)	77.0 (7.2)	76.8 (7.3)	<.001	0.09
Women^a^	2498 (46.8)	2603 (46.9)	2543 (45.4)	3422 (45.8)	.29	−0.02
Comorbidities						
Hypertension	3299 (61.8)	3632 (65.5)	3470 (62.0)	4886 (65.4)	<.001	−0.07
Diabetes	1339 (25.1)	1384 (25.0)	1410 (25.2)	1928 (25.8)	.67	−0.01
Bleeding disorder	1047 (19.6)	1041 (18.8)	982 (17.6)	1438 (19.2)	.03	0.01
COPD	858 (16.1)	947 (17.1)	838 (15.0)	1196 (16.0)	.03	0
Dementia	505 (9.5)	579 (10.4)	474 (8.5)	705 (9.4)	.006	0
Tobacco smoking	471 (8.8)	545 (9.8)	504 (9.0)	726 (9.7)	.16	−0.03
Functional dependency	444 (8.3)	554 (10.0)	499 (8.9)	748 (10.0)	.002	−0.06
Mechanism of injury						
Fall	3823 (71.6)	3949 (71.2)	3656 (65.3)	4774 (63.9)	<.001	−0.10
Transportation	1060 (19.9)	1191 (21.5)	1424 (25.4)	2086 (27.9)		
Other or unspecified	453 (8.5)	408 (7.4)	517 (9.2)	610 (8.2)		
Arrived as transfer	902 (16.9)	1221 (22.0)	1245 (22.2)	2333 (31.2)	<.001	−0.33
Fractured ribs, No.						
1	746 (14.0)	804 (14.5)	692 (12.4)	829 (11.1)	<.001	0
2-3	1404 (26.3)	1320 (23.8)	1346 (24.0)	1958 (26.2)		
4-7	980 (18.4)	1091 (19.7)	1175 (21.0)	1581 (21.2)		
≥8	130 (2.4)	161 (2.9)	158 (2.8)	247 (3.3)		
Multiple	1959 (36.7)	1972 (35.5)	2097 (37.5)	2754 (36.9)		
Not reported	117 (2.2)	200 (3.6)	129 (2.3)	101 (1.4)		
Flail chest	151 (2.8)	172 (3.1)	186 (3.3)	321 (4.3)	<.001	−0.08
Sternal fracture	281 (5.3)	340 (6.1)	395 (7.1)	566 (7.6)	<.001	−0.09
Pneumothorax	1317 (24.7)	1413 (25.5)	1477 (26.4)	1934 (25.9)	.21	−0.03
Hemothorax	651 (12.2)	740 (13.3)	728 (13.0)	1087 (14.6)	.001	−0.07
Lung contusion	475 (8.9)	540 (9.7)	579 (10.3)	817 (10.9)	.001	−0.07
ISS, mean (SD)						
All patients^b^	7.7 (3.4)	7.7 (3.6)	7.9 (3.5)	8.2 (3.5)	<.001	−0.15
Patients admitted to ICU^c^	9.2 (3.7)	9.0 (3.5)	9.1 (3.7)	9.0 (3.4)	.87	0.03
AIS thorax, mean (SD)						
All patients	2.6 (0.7)	2.6 (0.8)	2.6 (0.8)	2.6 (0.8)	<.001	−0.05
Patients admitted to ICU^d^	2.8 (0.7)	2.8 (0.7)	2.7 (0.8)	2.8 (0.7)	.10	0.10
Other minor injury, ie, AIS = 1						
Head	452 (8.5)	465 (8.4)	453 (8.1)	573 (7.7)	.33	0.03
Face	481 (9.0)	500 (9.0)	549 (9.8)	754 (10.1)	.08	−0.04
Neck	32 (0.6)	42 (0.8)	34 (0.6)	61 (0.8)	.37	−0.02
Abdomen	312 (5.8)	347 (6.2)	324 (5.8)	469 (6.3)	.54	−0.02
Spine	43 (0.8)	62 (1.1)	43 (0.8)	45 (0.6)	.01	0.02
Extremity						
Upper	807 (15.1)	826 (14.9)	825 (14.7)	1115 (14.9)	.96	0
Lower	646 (12.1)	633 (11.4)	604 (10.8)	963 (12.9)	.002	−0.02
External	301 (5.6)	287 (5.2)	291 (5.2)	325 (4.4)	.008	0.06

Abbreviations: AIS, Abbreviated Injury Scale; COPD, chronic obstructive pulmonary disease; ICU, intensive care unit; ISS, Injury Severity Score.

^a^ Sex missing for 2 patients in quartile 1 and 1 patient in quartile 4.

^b^ ISS missing for 5, 5, 63, and 59 patients in quartiles 1, 2, 3, and 4, respectively.

^c^ ISS missing for 0 of 203, 1 of 622 (0.2%), 13 of 1360 (1.0%), and 45 of 3738 patients (1.2%) in quartiles 1, 2, 3, and 4, respectively.

^d^ AIS thorax score available for all 203, 622, 1360, and 3738 patients in quartiles 1, 2, 3, and 4.

The most common comorbid conditions included hypertension, diabetes, bleeding disorders, and chronic obstructive pulmonary disease (Table 1). The most frequent mechanism of injury was a fall, followed by transportation-related injury. Approximately one-quarter of patients had 2-3 fractured ribs (6028 [25.2%]), but for more than one-third of patients, we could not characterize the number of fractured ribs from the NTDB data more specifically than multiple (8781 [36.7%]). Other than the thorax, the most common body regions with a minor injury (AIS = 1) were the upper (3573 [14.9%]) and lower (2846 [11.9%]) extremities. Most patients had initial vital signs within reference ranges (eTable 3 in the Supplement).

Greater ICU use was associated with increased volume of older patients with isolated rib fractures: hospitals in quartile 1 had a mean (SD) of 37 (26) such patients during the study period, while hospitals in quartile 4 had a mean (SD) of 52 (39) such patients (Table 2). More hospitals in the highest quartile of ICU use were level I trauma centers, university-affiliated teaching hospitals, hospitals with more than 500 beds, and hospitals located in the southern (52 [36.4%]) and western (40 [28.0%]) regions.

**Table 2.** Baseline Characteristics of the 573 Hospitals by Quartile of Hospital-Level ICU Use

Hospital characteristic	Hospital-level proportion of older patients with isolated rib fractures admitted to an ICU, No. (%)				*P* value	Standardized difference, quartiles 1 and 4
	Quartile 1, 0%-7.3% (n = 143)	Quartile 2, 7.4%-16.6% (n = 143)	Quartile 3, 16.7%-32.0% (n = 144)	Quartile 4, 32.1%-91.9% (n = 143)		
Eligible hospitalizations, mean (SD)	37.3 (26.3)	38.8 (27.5)	38.9 (31.1)	52.2 (39.0)	<.001	−0.45
Trauma center level						
I	29 (20.3)	37 (25.9)	45 (31.2)	80 (55.9)	<.001	0.67
II	63 (44.1)	66 (46.2)	70 (48.6)	47 (32.9)		
III	41 (28.7)	33 (23.1)	25 (17.4)	11 (7.7)		
IV	1 (0.7)	2 (1.4)	1 (0.7)	0 (0)		
Not reported	9 (6.3)	5 (3.5)	3 (2.1)	5 (3.5)		
Teaching status						
Community	69 (48.2)	77 (53.8)	61 (42.4)	53 (37.1)	<.001	−0.46
Nonteaching	44 (30.8)	30 (21.0)	45 (31.2)	19 (13.3)		
University	30 (21.0)	36 (25.2)	38 (26.4)	71 (49.6)		
Region						
Northeast	36 (25.2)	33 (23.1)	18 (12.5)	24 (16.8)	<.001	−0.01
South	24 (16.8)	48 (33.6)	51 (35.4)	52 (36.4)		
Midwest	52 (36.4)	37 (25.9)	36 (25.0)	27 (18.9)		
West	28 (19.6)	25 (17.5)	39 (27.1)	40 (28.0)		
Not reported	3 (2.1)	0	0	0		
Adult beds						
>500	29 (20.3)	35 (24.5)	29 (20.1)	57 (39.9)	.002	0.50
351-500	30 (21.0)	30 (21.0)	40 (27.8)	36 (25.2)		
251-350	35 (24.5)	41 (28.7)	39 (27.1)	27 (18. 9)		
101-250	43 (30.1)	34 (23.8)	33 (22.9)	19 (13.3)		
1-100	6 (4.2)	3 (2.1)	1 (0.7)	3 (2.1)		
Not reported	0	0	2 (1.4)	1 (0.7)		
ICU trauma beds						
>35	24 (16.8)	28 (19.6)	34 (23.6)	54 (37.8)	.006	−0.46
26-35	18 (12.6)	26 (18.2)	30 (20.3)	19 (13.3)		
16-25	53 (37.1)	50 (35.0)	50 (34.7)	41 (28.7)		
11-15	29 (20.3)	21 (14.7)	17 (11.8)	19 (13.3)		
1-10	10 (7.0)	12 (8.4)	10 (6.9)	7 (4.9)		
0	8 (5.6)	6 (4.2)	1 (0.7)	3 (2.1)		
Not reported	1 (0.7)	0	2 (1.4)	0		
Stepdown unit present	112 (78.3)	114 (79.7)	110 (76.4)	107 (74.8)	.77	0.08
Proportion of trauma patients age ≥65 y, %^a^						
50-100	21 (14.7)	14 (9.8)	11 (7.6)	6 (4.2)	<.001	0.89
40 to <50	46 (32.2)	27 (18.9)	31 (21.5)	16 (11.2)		
30 to <40	47 (32.9)	55 (38.5)	37 (25.7)	36 (25.2)		
20 to <30	21 (14.7)	39 (27.3)	41 (28.5)	52 (36.4)		
<20%	8 (5.6)	8 (5.6)	24 (16.7)	33 (23.1)		
Proportion of trauma patients with blunt mechanism, %^b^						
95-100	15 (10.5)	13 (9.1)	10 (6.9)	10 (7.0)	<.001	0.81
90 to <95	85 (59.4)	57 (39.9)	60 (41.7)	34 (23.8)		
85 to <90	28 (19.6)	46 (32.2)	44 (30.6)	41 (28.7)		
80 to <85	7 (4.9)	19 (13.3)	15 (10.4)	22 (15.4)		
<80%	8 (5.6)	8 (5.6)	15 (10.4)	36 (25.2)		

Abbreviations: ICU, intensive care unit; NTDB, National Trauma Data Bank.

^a^ The proportion of all trauma patients per hospital in the NTDB during 2015 to 2016 who were aged 65 years or older.

^b^ The proportion of all trauma patients per hospital in the NTDB during 2015-2016 who had a blunt mechanism of injury.

The composite outcome occurred in 787 patients (3.3%), with unplanned intubation in 317 (1.3%), pneumonia in 180 (0.8%), and death in 451 (1.9%). Using generalized linear mixed models, compared with the lowest quartile of ICU use, all quartiles of greater ICU use were associated with increased unadjusted odds of the composite adverse outcome (Table 3). However, with adjustment for propensity scores, quartiles 2 and 3 were not associated with an increase in the composite outcome (quartile 2: OR, 1.17; 95% CI, 0.90-1.52; quartile 3: OR, 0.99; 95% CI, 0.76-1.28), and quartile 4 was associated with decreased odds of the composite outcome (OR, 0.71; 95% CI, 0.55-0.92) (Table 3). Relative to quartile 1, there was a significantly greater adjusted odds of death in quartile 2 (OR, 1.42; 95% CI, 1.03-1.97) and a significantly lower adjusted odds of pneumonia in quartiles 2 (OR, 0.61; 95% CI, 0.37-0.997) and 4 (OR, 0.54; 95% CI, 0.34-0.85). There was no significant difference in the odds of unplanned intubation, adult respiratory distress syndrome, or unplanned ICU transfer across quartiles. The sensitivity analyses generally confirmed the association of improved outcomes with greatest quartile of ICU use; however, inclusion of telemetry or stepdown units in the definition of ICU admission reversed this association (Table 4).

**Table 3.** Associations Between Hospital-Level ICU Use and Outcomes Using Generalized Linear Mixed Models

Outcome	Quartile 1 (n = 5336)	Quartile 2 (n = 5548)	Quartile 3 (n = 5597)	Quartile 4 (n = 7470)
Composite				
No. (%)	131 (2.5)	196 (3.5)	198 (3.5)	262 (3.5)
Unadjusted OR (95% CI)	1 [Reference]	1.42 (1.12-1.81)	1.45 (1.14-1.84)	1.43 (1.14-1.80)
Adjusted OR (95% CI)^a^	1 [Reference]	1.17 (0.90-1.52)	0.99 (0.76-1.28)	0.71 (0.55-0.92)
Death				
No. (%)	66 (1.2)	117 (2.1)	106 (1.9)	162 (2.2)
Unadjusted OR (95% CI)	1 [Reference]	1.70 (1.25-2.33)	1.53 (1.12-2.11)	1.75 (1.30-2.36)
Adjusted OR (95% CI)^a^	1 [Reference]	1.42 (1.03-1.97)	1.06 (0.76-1.48)	0.91 (0.66-1.26)
Pneumonia				
No. (%)	44 (0.8)	33 (0.6)	45 (0.8)	58 (0.8)
Unadjusted OR (95% CI)	1 [Reference]	0.71 (0.44-1.14)	0.99 (0.63-1.55)	0.93 (0.61-1.42)
Adjusted OR (95% CI)^a^	1 [Reference]	0.61 (0.37-0.997)	0.73 (0.46-1.17)	0.54 (0.34-0.85)
Unplanned intubation				
No. (%)	49 (0.9)	75 (1.4)	79 (1.4)	114 (1.5)
Unadjusted OR (95% CI)	1 [Reference]	1.43 (0.96-2.14)	1.53 (1.03-2.28)	1.67 (1.14-2.43)
Adjusted OR (95% CI)^a^	1 [Reference]	1.13 (0.74-1.74)	0.97 (0.63-1.50)	0.75 (0.49-1.13)
ARDS				
No. (%)	18 (0.3)	21 (0.4)	16 (0.3)	32 (0.4)
Unadjusted OR (95% CI)	1 [Reference]	1.09 (0.52-2.25)	0.83 (0.38-1.80)	1.14 (0.57-2.27)
Adjusted OR (95% CI)^a^	1 [Reference]	0.90 (0.43-1.90)	0.54 (0.24-1.19)	0.51 (0.24-1.06)
Unplanned ICU transfer				
No. (%)	96 (1.8)	127 (2.3)	123 (2.2)	214 (2.9)
Unadjusted OR (95% CI)	1 [Reference]	1.19 (0.86-1.65)	1.20 (0.87-1.67)	1.61 (1.19-2.18)
Adjusted OR (95% CI)^a^	1 [Reference]	1.02 (0.72-1.42)	0.86 (0.61-1.20)	0.89 (0.65-1.23)

Abbreviations: ARDS, adult respiratory distress syndrome; ICU, intensive care unit; OR, odds ratio.

^a^ ORs are adjusted for propensity scores based on patient-level and hospital-level factors associated with ICU admission (eAppendix 2 in the Supplement).

**Table 4.** Sensitivity Analyses Using Alternative Definitions of the Study Cohort, Exposure Status, and Outcome

Sensitivity analysis	Quartile 1	Quartile 2	Quartile 3	Quartile 4
Expanding definition of ICU to include admission to telemetry or stepdown units				
Events/total (%)	125/5034 (2.5)	167/5545 (3.0)	213/6408 (3.3)	282/6964 (4.0)
aOR (95% CI)^a^	1 [Reference]	1.12 (0.87-1.44)	1.17 (0.91-1.50)	1.39 (1.09-1.78)
Expanding cohort to include patients with nonthoracic injuries with AIS score ≤2				
Events/total (%)	294/8778 (3.3)	452/10 539 (4.3)	525/11 561 (4.5)	706/15 726 (4.5)
aOR (95% CI)^a^	1 [Reference]	1.06 (0.87-1.31)	0.92 (0.75-1.13)	0.71 (0.58-0.86)
Including patients admitted to observation status, considered non-ICU				
Events/total (%)	139/5522 (2.5)	194/5906 (3.3)	194/5590 (3.5)	268/7910 (3.4)
aOR (95% CI)^a^	1 [Reference]	1.07 (0.83-1.38)	0.99 (0.76-1.28)	0.69 (0.54-0.89)
Restricting cohort to patients with ≥2 fractured ribs				
Events/total (%)	112/4205 (2.7)	182/4689 (3.9)	180/4832 (3.7)	234/6348 (3.7)
aOR (95% CI)^a^	1 [Reference]	1.20 (0.92-1.58)	0.96 (0.73-1.27)	0.73 (0.55-0.95)
Restricting cohort to patients with ≥3 fractured ribs				
Events/total (%)	98/3565 (2.7)	147/3438 (4.3)	150/4058 (3.7)	212/5076 (4.2)
aOR (95% CI)^a^	1 [Reference]	1.35 (1.01-1.81)	0.98 (0.73-1.31)	0.79 (0.60-1.04)
Restricting cohort to hospitals with ≥20 eligible patients during the study period				
Events/total (%)	117/4838 (2.4)	193/5142 (3.8)	183/5365 (3.4)	234/6522 (3.6)
aOR (95% CI)^a^	1 [Reference]	1.22 (0.92-1.60)	0.94 (0.71-1.24)	0.71 (0.54-0.94)
Expanding definition of the composite outcome to include discharge to hospice				
Events/total	177/5336 (3.3)	244/5548 (4.4)	249/5597 (4.4)	338/7470 (4.5)
aOR (95% CI)^a^	1 [Reference]	1.10 (0.87-1.39)	0.96 (0.76-1.22)	0.74 (0.59-0.93)

Abbreviations: AIS, Abbreviated Injury Scale; aOR, adjusted odds ratio; ICU, intensive care unit.

^a^ Adjusted for propensity scores based on patient-level and hospital-level factors associated with ICU admission (eAppendix 2 in the Supplement).

Relative to quartile 1, greater hospital-level ICU use was associated with longer patient-level ICU length of stay by an adjusted factor of 1.6 (95% CI, 1.4-2.0) for quartile 2, 2.4 (95% CI, 2.0-2.8) for quartile 3, and 2.9 (95% CI, 2.4-3.4) for quartile 4. There was no significant association between ICU use and ventilator days. Relative to quartile 1, ventilator days for quartiles 2, 3, and 4 increased by adjusted factors of 1.3 (95% CI, 0.9-1.9), 1.4 (95% CI, 0.97-2.1), and 1.1 (95% CI, 0.8-1.7), respectively.

## Discussion

This study suggests that there is wide national variability among trauma centers in ICU admission for older patients with isolated rib fractures, ranging from none to most such patients. We found that, relative to hospitals in the lowest quartile of ICU use, patients at hospitals in the highest quartile had 29% lower adjusted odds of the composite outcome. Assuming that the likelihood of the outcome at hospitals with low ICU use is 2.5%, this corresponds to a number needed to treat (NNT) of 140 patients treated at a hospital with high ICU use (as opposed to a hospital with low ICU use) to avoid 1 unplanned intubation, occurrence of pneumonia, or death.^9^

Among older patients with rib fractures, early studies estimated rates of pneumonia and death as high as 34% and 14%, respectively.^1^ We observed pneumonia in 0.7% of patients and death in 1.9%. Among patients with 1 to 2 rib fractures, Ho et al^3^ similarly observed pneumonia in 1.6%. The low incidence of pneumonia and death in our study is likely because we excluded patients with other significant injuries or another indication for ICU admission (eg, GCS score <9, intubation).

We found that hospitals with the greatest ICU use were associated with improved inpatient outcomes. Two prior studies of older patients with blunt thoracic trauma found ICU admission to be associated with fewer complications^10^ or decreased mortality,^8^ whereas another identified increased mortality.^11^ None of these studies specifically evaluated patients with isolated rib fractures. Among patients at our center, we observed that only 25% of older patients with isolated rib fractures experienced an intervention or event suggestive of requiring ICU-level care,^12^ so consistent with the large NNT we estimated, any putative benefits of ICU admission may apply to a relatively small subpopulation of all older patients with isolated rib fractures. Several studies have retrospectively identified patient and injury characteristics that increase the likelihood of poor outcomes,^13,14,15,16^ although few have focused on older patients with isolated rib fractures. Characteristics that may more specifically identify high-risk patients include those with particular anatomic characteristics of rib fractures^14,17^ or impaired performance with pulmonary function testing.^18^

Variable ICU use has been demonstrated for other diagnoses, including diabetic ketoacidosis, pulmonary embolism, traumatic subarachnoid hemorrhage, abdominal solid organ injuries, pneumonia, and heart failure.^19,20,21,22,23,24^ Admon et al^25^ have argued that ICU use is a hospital-specific characteristic, correlated across several disease diagnoses and thus may be independent of a specific patient population. One recent cluster randomized trial evaluating systematic ICU admission of patients with defined critical conditions (as opposed to an existing standard practice of more selective ICU admission) concluded that systematic ICU admission did not reduce 6-month mortality.^26^ However, differences in the study setting and patient population may explain why the findings differed from our study.

The sensitivity analyses we performed generally confirmed an association of improved outcomes with greater ICU use. Based on the WTA guidelines,^5^ we specifically evaluated whether restricting the study cohort to patients with at least 2 or at least 3 fractured ribs yielded different results or an augmented association, but the results did not substantially change, suggesting that the number of fractured ribs is not a key determinant of the putative benefits of ICU care. The lack of an association in the sensitivity analysis that defined ICU use to include telemetry or stepdown status suggests that telemetry and stepdown units may not offer the same benefits as ICUs.

We evaluated unplanned ICU transfer as a potential additional indicator that admission to an ICU helped prevent patient deterioration short of resulting in unplanned intubation, pneumonia, or death. Patients with rib fractures are among the most common patients who return to the ICU, typically because of respiratory failure.^27^ Although we did not observe a decrease in unplanned ICU transfer associated with high ICU admission from the ED, we also did not observe an increase. Thus, it seems implausible that patients at hospitals with high ICU use avoided developing pneumonia or requiring intubation only by robust rescue processes.

The aging US population is profoundly affecting its health care system. Among Medicare fee-for-service beneficiaries, 29% spent time in an ICU during their last 30 days of life.^28^

### Strengths and Limitations

The strengths of our study include the narrow definition of the study population such that ICU admission was plausibly intended to prevent pulmonary complications, the wide variability in exposure status (hospital-level ICU admission rates) we observed, the relevance of the outcomes to older patients with chest wall injuries, accounting for the hierarchical nature of the data, adjusting for a variety of patient-level and hospital-level potential confounding factors, and consideration of hospital-level ICU use as a possible preference instrument. Nonetheless, our study has important limitations. While we thoroughly evaluated the available information in the NTDB, there may be residual confounding from unrecorded patient-level or hospital-level characteristics that could be associated with greater ICU use (eg, less use of the ICU for palliation or increased availability of respiratory therapists or geriatricians). The NTDB does not contain any information about (nor does it allow linkage to determine) which hospitals use an algorithm to determine ICU admission (and, if so, what criteria they use) or clinical guidelines to standardize pain management and respiratory therapy. The association of such factors with ICU use is difficult to predict but could have biased the observed association in either direction (favoring or disfavoring high ICU use) with large or small magnitude. Our study is also limited because the NTDB only contains information about the index hospitalization, so we cannot draw conclusions about hospital readmissions or other postdischarge outcomes, including death. The best we could do in this regard was to expand the definition of the composite outcome to include discharge to hospice in a sensitivity analysis.

## Conclusions

In this study, greater hospital-level ICU use was associated with better outcomes among older patients with isolated rib fractures, and it may be warranted for hospitals with low ICU use to admit more such patients to an ICU. ICU beds are a limited resource, and their use ideally should be informed by evidence. Future studies might focus on confirming whether ICU care appears to be beneficial, using other data sets and methods (eg, instrumental variable analysis, if applicable) and discerning whether ICU use benefits specific subgroups of patients with chest wall injuries. Additionally, longitudinal studies could help evaluate morbidity and mortality after discharge and address the potential cost-effectiveness of ICU admission.

## Notes

Supplement. **eAppendix 1. **AIS Predot and *International Classification of Diseases* Codes for Rib Fractures **eAppendix 2. **Propensity Scoring and Instrumental Variable Analysis Methods **eTable 1. **Thoracic AIS Predot Codes (1998 Update of 1990 Version) Used for Exclusion Criteria in the 2015 NTDB Data Set **eTable 2. **Thoracic AIS Predot Codes (2008 Update of the 2005 Version) Used for Exclusion Criteria in the 2016 NTDB Data Set **eTable 3. **Baseline Physiologic Characteristics **eTable 4. **Strength of Instrumental Variables Based on Hospital ICU Utilization in Predicting ICU Admission for Individual Patients **eTable 5. **Covariate Balance by Exposure and Instrument Status **eTable 6. **Patient-Level and Hospital-Level Characteristics Incorporated Into the Propensity Scores **eFigure. **Rank Order Plot of Hospital-Level ICU Use
