# Supplementary material for: Association of Hospital-Level Intensive Care Unit Use and Outcomes in Older Patients With Isolated Rib Fractures
Source: JAMA Netw Open. 2020 Nov 19;3(11):e2026500. doi: 10.1001/jamanetworkopen.2020.26500 (PMC7677756; doi:10.1001/jamanetworkopen.2020.26500)
Supplement: Supplement. — eAppendix 1. AIS Predot and International Classification of Diseases Codes for Rib Fractures eAppendix 2. Propensity Scoring and Instrumental Variable Analysis Methods eTable 1. Thoracic AIS Predot Codes (1998 Update of 1990 Version) Used for Exclusion Criteria in the 2015 NTDB Data Set eTable 2. Thoracic AIS Predot Codes (2008 Update of the 2005 Version) Used for Exclusion Criteria in the 2016 NTDB Data Set eTable 3. Baseline Physiologic Characteristics eTable 4. Strength of Instrumental Variables Based on Hospital ICU Utilization in Predicting ICU Admission for Individual Patients eTable 5. Covariate Balance by Exposure and Instrument Status eTable 6. Patient-Level and Hospital-Level Characteristics Incorporated Into the Propensity Scores eFigure. Rank Order Plot of Hospital-Level ICU Use [file jamanetwopen-e2026500-s001.pdf]

Supplemental Online Content

Bowman JA, Nuño M, Jurkovich GJ, Utter GH. Association of hospital-level intensive care unit use and outcomes in older patients with isolated rib fractures. *JAMA Netw Open*. 2020;3(11):e2026500. doi:10.1001/jamanetworkopen.2020.26500

eAppendix 1. AIS Predot and *International Classification of Diseases* Codes for Rib Fractures
eAppendix 2. Propensity Scoring and Instrumental Variable Analysis Methods
eTable 1. Thoracic AIS Predot Codes (1998 Update of 1990 Version) Used for Exclusion Criteria in the 2015 NTDB Data Set
eTable 2. Thoracic AIS Predot Codes (2008 Update of the 2005 Version) Used for Exclusion Criteria in the 2016 NTDB Data Set
eTable 3. Baseline Physiologic Characteristics
eTable 4. Strength of Instrumental Variables Based on Hospital ICU Utilization in Predicting ICU Admission for Individual Patients
eTable 5. Covariate Balance by Exposure and Instrument Status
eTable 6. Patient-Level and Hospital-Level Characteristics Incorporated Into the Propensity Scores
eFigure. Rank Order Plot of Hospital-Level ICU Use

This supplemental material has been provided by the authors to give readers additional information about their work.

eAppendix 1. AIS Predot and International Classification of Diseases Codes for Rib Fractures

Codes defining rib fractures

AIS "predot" codes (1998 update of the 1990 version) used for the 2015 NTDB dataset

450210	Multiple rib fractures, NFS
450211	Multiple rib fractures, NFS, with hemo-/pneumothorax
450212	1 rib fracture
450214	1 rib fracture, with hemo-/pneumothorax (OIS Grade I)
450220	2-3 rib fractures any location or multiple fractures of single rib, with stable chest or NFS (OIS Grade I, II, III)
450222	2-3 rib fractures any location or multiple fractures of single rib, with stable chest or NFS (OIS Grade I, II, III), with hemo-/pneumothorax
450230	>3 rib fractures on one side and no more than 3 ribs on the other side, stable chest or NFS (OIS Grade I, II, III)
450232	>3 rib fractures on one side and no more than 3 ribs on the other side, stable chest or NFS (OIS Grade I, II, III), with hemo-/pneumothorax
450240	>3 rib fractures on each of two sides, with stable chest or NFS
450242	>3 rib fractures on each of two sides, with stable chest or NFS, with hemo-/pneumothorax
450250	Open/displaced/comminuted (any or combination) ≥1 rib fracture
450252	Open/displaced/comminuted (any or combination) ≥1 rib fracture, with hemo-/pneumothorax
450260	Flail chest (unstable chest wall, paradoxical chest movement), unilateral or NFS (OIS Grade III or IV)
450262	Flail chest (unstable chest wall, paradoxical chest movement), unilateral or NFS, without lung contusion (OIS Grade III or IV)
450264	Flail chest (unstable chest wall, paradoxical chest movement), unilateral or NFS, with lung contusion (OIS Grade III or IV)
450266	Flail chest (unstable chest wall, paradoxical chest movement), bilateral (OIS Grade V)

AIS "predot" codes (2008 update of the 2005 version) used for the 2016 NTDB dataset

450200	Rib fracture(s) without flail, any location unilateral or bilateral NFS
450201	Rib fracture, 1 rib (OIS Grade I)
450202	Rib fractures, 2 ribs (OIS Grade I)
450203	Rib fractures, ≥ 3 ribs (OIS Grade II)
450209	Rib fractures with flail, NFS
450210	Multiple rib fractures NFS
450211	Rib fractures with unilateral flail chest, NFS (OIS Grade I)
450212	Rib fractures with unilateral flail chest, 3-5 flail ribs (OIS Grade IV)
450213	Rib fractures with unilateral flail chest, >5 flail ribs (OIS Grade IV)
450214	Rib fractures with bilateral flail chest (OIS Grade V)

ICD-9-CM diagnosis codes

807.00	Fracture of rib(s), unspecified, closed
807.01	Fracture of one rib, closed
807.02	Fracture of two ribs, closed
807.03	Fracture of three ribs, closed
807.04	Fracture of four ribs, closed
807.05	Fracture of five ribs, closed
807.06	Fracture of six ribs, closed
807.07	Fracture of seven ribs, closed
807.08	Fracture of eight or more ribs, closed
807.09	Fracture of multiple ribs, unspecified, closed
807.10	Fracture of rib(s), unspecified, open
807.11	Fracture of one rib, open
807.12	Fracture of two ribs, open

807.13	Fracture of three ribs, open
807.14	Fracture of four ribs, open
807.15	Fracture of five ribs, open
807.16	Fracture of six ribs, open
807.17	Fracture of seven ribs, open
807.18	Fracture of eight or more ribs, open
807.19	Fracture of multiple ribs, unspecified, open
807.4	Flail chest

<u>ICD-10-CM diagnosis codes</u>

S22.31XA	Fracture of one rib, right side, initial encounter for closed fracture
S22.31XB	Fracture of one rib, right side, initial encounter for open fracture
S22.32XA	Fracture of one rib, left side, initial encounter for closed fracture
S22.32XB	Fracture of one rib, left side, initial encounter for open fracture
S22.39XA	Fracture of one rib, unspecified side, initial encounter for closed fracture
S22.39XB	Fracture of one rib, unspecified side, initial encounter for open fracture
S22.41XA	Multiple fractures of ribs, right side, initial encounter for closed fracture
S22.41XB	Multiple fractures of ribs, right side, initial encounter for open fracture
S22.42XA	Multiple fractures of ribs, left side, initial encounter for closed fracture
S22.42XB	Multiple fractures of ribs, left side, initial encounter for open fracture
S22.43XA	Multiple fractures of ribs, bilateral, initial encounter for closed fracture
S22.43XB	Multiple fractures of ribs, bilateral, initial encounter for open fracture
S22.49XA	Multiple fractures of ribs, unspecified side, initial encounter for closed fracture
S22.49XB	Multiple fractures of ribs, unspecified side, initial encounter for open fracture
S22.5XXA	Flail chest, initial encounter for closed fracture
S22.5XXB	Flail chest, initial encounter for open fracture

<u>ICD-10-CA diagnosis codes</u>

S22.300	Fracture of rib, closed
S22.301	Fracture of rib, open
S22.400	Multiple fractures of 2-4 ribs, closed
S22.401	Multiple fractures of 2-4 ribs, open
S22.410	Multiple fractures of 5 or more ribs, closed
S22.411	Multiple fractures of 5 or more ribs, open
S22.490	Multiple fractures of unspecified number of ribs, closed
S22.491	Multiple fractures of unspecified number of ribs, open
S22.500	Flail chest, closed
S22.501	Flail chest, open

© 2020 Bowman JA et al. *JAMA Network Open.*

eAppendix 2. Propensity Scoring and Instrumental Variable Analysis Methods

Defining instruments: Mixed effects logistic regression models

As a strategy to address the possibility of confounding, we evaluated the validity of instrumental variable (IV) methods based on hospital ICU use. To estimate hospital-specific ICU use adjusted for case-mix and hospital characteristics, we used mixed-effects logistic regression models. We modeled hospital identity as a random effect and patient and hospital characteristics as fixed effects. To minimize the likelihood of model convergence problems, we used propensity scores rather than individual variables to adjust for all available patient and hospital characteristics. We derived propensity scores as described below (see "Propensity scoring"), but centered the propensity scores on the mean and estimated the probability that the average patient, defined as the patient with a mean propensity score, would be admitted to the ICU in each hospital. We created IV measures by assessing the distribution of random intercepts from the fully adjusted mixed-effects model and derived median, tertile, and quartile cutoff values. We then uniquely assigned each patient to median, tertile, and quartile groups using these cutoffs.

The multilevel analysis described above is a general method of analyzing data with a hierarchical or clustered structure. Multilevel models allow clinical decisions—such as the decision to admit patients to an ICU—to depend on patient, physician, and hospital fixed and random effects in complex ways, and have previously been applied for the profiling of health care providers. Our data set consists of two distinct levels: patient- and hospital-level information. We specified a mixed effects logistic regression model that allowed the patient-level outcome (being admitted to the ICU) to depend on patient and hospital fixed effects (embedded in the propensity score) as well as hospital random effects:

$$\ln\left(\frac{p_{ij}}{1-p_{ij}}\right) = \beta_0 + \beta_1 * PS\_centered_{ij} + b_j$$

p_{ij} : i^{th} patient of the jth hospital's probability of being admitted to the ICU
β_1 : coefficient associated with the patient-level propensity score, centered on the mean
β_0 : marginal (averaged across hospitals) probability of ICU admission for a patient with the mean propensity score (PS centered=0)
b_j : hospital-level random intercept
b_j $\sim N(0,\sigma_b^2)$, where σ_b^2 is the between-hospital variance and represents the hospital-specific deviation from β_0

The parameter of interest for the description of the variation in ICU tendency is σ_b^2. The hospital-specific probability of ICU utilization for the "average" or "typical" patient in the study is given by:

$$\frac{\exp(\beta_0 + b_j)}{1 + \exp(\beta_0 + b_j)}$$

The interval that contains the ICU preference probability for the 95% hospitals in the center of the range is given by:

$$\left(\frac{\exp(\beta_0 - 1.96\sigma_b)}{1+\exp(\beta_0 - 1.96\sigma_b)};\frac{\exp(\beta_0 + 1.96\sigma_b)}{1+\exp(\beta_0 + 1.96\sigma_b)}\right)$$

We assessed the plausibility of IV assumptions by: (1) computing the square of the semi-partial correlation between the instrument and ICU treatment, conditional on the other covariates in the model, to assess instrument strength; (2) assessing whether the instrument provided a natural experiment in treatment choice by evaluating whether the distributions of covariates between patients admitted to hospitals with the highest and lowest quantile groups were similar; and (3) examining the association between the IV and characteristics of hospitals associated with high-quality care (e.g., trauma center level) to evaluate empirically whether the IV might be related to the outcome (other than through exposure), thus violating the exogeneity assumption.

© 2020 Bowman JA et al. *JAMA Network Open.*

We sought to estimate the risk for each outcome associated with ICU admission using a two-stage prediction substitution generalized logistic regression model. We sought to assess whether each version of the instrument (i.e., median, tertile, quartile) influenced the observed associations.

In our analyses, instrumental variables based on hospital ICU utilization practices were strongly associated with actual ICU admission, explaining 0.05 to 10.2% of the variance in exposure in adjusted analysis (eTable 4). Patient characteristics were distributed similarly across quantiles based on IVs (eTable 5). However, the IVs were associated with other characteristics of hospitals reflecting or known to have a relationship with high-quality care (e.g., trauma center level, hospital size, and the occurrence of any complication), violating the exogeneity assumption. Because the exogeneity assumption is vital to the premise of an instrumental variable, violation of this assumption meant that this approach was not valid for the data we analyzed. Therefore, we present analyses using generalized linear mixed models instead.

Propensity scoring

For the generalized linear mixed model analyses, we used propensity scores to adjust for potential confounding factors. Because inclusion of a large number of covariates can lead to convergence problems in multilevel models with relatively small cluster sizes, we adjusted for all patient and hospital characteristics available in the study dataset (eTable 6) with summary propensity scores rather than individual variables. We derived propensity scores from predicted probabilities of exposure (ICU admission versus non-ICU admission) estimated via a logistic regression model. We incorporated propensity scores into the generalized linear mixed models as simple probabilities, ranging from 0 to 1, without stratification or matching.

© 2020 Bowman JA et al. *JAMA Network Open.*

eTable 1. Thoracic AIS Predot Codes (1998 Update of 1990 Version) Used for Exclusion Criteria in the 2015 NTDB Data Set

AIS "predot" code	AIS Score	Description
413000	6	Bilateral destruction of skeletal, vascular, organ and tissue systems ("crush" injury)
415000	4	Open ("sucking") chest wound (OIS Grade IV)
416006	3	Penetrating injury with blood loss >20% by volume
416008	3	Penetrating injury with hemo-/pneumothorax except tension pneumothorax
410606	3	Skin/subcutaneous/muscles/chest wall: laceration, blood loss >20% by volume
410806	3	Skin/subcutaneous/muscles/chest wall: avulsion, blood loss >20% by volume
420299	4	Aorta, thoracic, NFS
420202	4	Aorta, thoracic: intimal tear, no disruption
420204	5	Aorta, thoracic: intimal tear, no disruption, with aortic valve involvement
420206	4	Aorta, thoracic: laceration (perforation, puncture) NFS
420208	4	Aorta, thoracic: laceration (perforation, puncture): minor
420210	5	Aorta, thoracic: laceration (perforation, puncture): major
420212	5	Aorta, thoracic: laceration (perforation, puncture): major, with aortic root or valve involvement
420216	5	Aorta, thoracic: laceration (perforation, puncture): major, with hemorrhage confined to mediastinum
420218	6	Aorta, thoracic: laceration (perforation, puncture): major, with hemorrhage not confined to mediastinum
420499	3	Brachiocephalic (innominate) artery NFS
420402	3	Brachiocephalic (innominate) artery: intimal tear, no disruption
420404	3	Brachiocephalic (innominate) artery: laceration (perforation, puncture) NFS
420406	3	Brachiocephalic (innominate) artery: laceration (perforation, puncture): minor
420408	4	Brachiocephalic (innominate) artery: laceration (perforation, puncture): major
420699	3	Brachiocephalic (innominate) vein NFS
420602	3	Brachiocephalic (innominate) vein: laceration (perforation, puncture) NFS
420604	3	Brachiocephalic (innominate) vein: laceration (perforation, puncture): minor
420606	4	Brachiocephalic (innominate) vein: laceration (perforation, puncture): major
420608	5	Brachiocephalic (innominate) vein: laceration (perforation, puncture): major, with air embolus right side
420800	5	Coronary artery laceration or thrombosis (left main, right main or left anterior descending artery; coronary sinus)
421099	3	Pulmonary artery NFS
421002	3	Pulmonary artery: intimal tear, no disruption
421004	3	Pulmonary artery: laceration (perforation, puncture) NFS
421006	3	Pulmonary artery: laceration (perforation, puncture): minor
421008	4	Pulmonary artery: laceration (perforation, puncture): major
421299	3	Pulmonary vein NFS
421202	3	Pulmonary vein: laceration (perforation, puncture) NFS
421204	3	Pulmonary vein: laceration (perforation, puncture): minor

© 2020 Bowman JA et al. *JAMA Network Open.*

eTable 1. Thoracic AIS Predot Codes (1998 Update of 1990 Version) Used for Exclusion Criteria in the 2015 NTDB Data Set (continued)

AIS "predot" code	AIS Score	Description
421206	4	Pulmonary vein: laceration (perforation, puncture): major
421499	3	Subclavian artery NFS
421402	3	Subclavian artery: intimal tear, no disruption
421404	3	Subclavian artery: laceration (perforation, puncture) NFS
421406	3	Subclavian artery: laceration (perforation, puncture): minor
421408	4	Subclavian artery: laceration (perforation, puncture): major
421699	3	Subclavian vein NFS
421602	3	Subclavian vein: laceration (perforation, puncture) NFS
421604	3	Subclavian vein: laceration (perforation, puncture): minor
421606	4	Subclavian vein: laceration (perforation, puncture): major
421899	3	Vena Cava, superior and thoracic portion of inferior, NFS
421802	3	Vena Cava, superior and thoracic portion of inferior: laceration (perforation, puncture) NFS
421804	3	Vena Cava, superior and thoracic portion of inferior: laceration (perforation, puncture): minor with or without thrombosis
421806	4	Vena Cava, superior and thoracic portion of inferior: laceration (perforation, puncture): major
421808	5	Vena Cava, superior and thoracic portion of inferior: laceration (perforation, puncture): major, with air embolus right side
422008	3	Other named arteries NFS (e.g., bronchial, esophageal, intercostal, internal mammary): laceration (perforation, puncture): major
422206	3	Other named veins NFS (e.g., azygos, bronchial, hemiazygos, intercostal, internal mammary, internal jugular): laceration (perforation, puncture): major
440208	3	Bronchus distal to main stem: laceration (puncture): perforation; full thickness but not complete transection
440210	4	Bronchus distal to main stem: laceration (puncture): complex; avulsion; rupture; transection
440212	3	Bronchus distal to main stem: fracture NFS
440214	3	Bronchus distal to main stem: fracture: simple
440216	4	Bronchus distal to main stem: fracture: major (with separation)
440400	5	Chordae tendinae laceration (rupture)
440604	3	Diaphragm: laceration
440606	4	Diaphragm: rupture with herniation
440804	3	Esophagus: laceration NFS
440806	3	Esophagus: laceration: no perforation; partial thickness; ≤50% circumference
440808	4	Esophagus: laceration: perforation; full thickness but not complete transection; >50% circumference
440810	5	Esophagus: laceration complex with tissue loss; avulsion; rupture; transection
441006	4	Heart (Myocardium): contusion (hematoma): major
441008	3	Heart (Myocardium): laceration NFS

© 2020 Bowman JA et al. *JAMA Network Open.*

eTable 1. Thoracic AIS Predot Codes (1998 Update of 1990 Version) Used for Exclusion Criteria in the 2015 NTDB Data Set (continued)

AIS "predot" code	AIS Score	Description
441010	3	Heart (Myocardium): laceration: no perforation, no chamber involvement
441012	5	Heart (Myocardium): laceration: perforation (ventricular or atrial with or without tamponade)
441014	6	Heart (Myocardium): laceration: perforation (ventricular or atrial with or without tamponade): complex or ventricular rupture
441016	6	Heart (Myocardium): laceration: perforation (ventricular or atrial with or without tamponade): multiple lacerations; >50% tissue loss of a chamber
441018	6	Heart (Myocardium): avulsion
441200	5	Intracardiac valve laceration (rupture)
441300	5	Interventricular or interatrial septum laceration (rupture)
441499	3	Lung NFS
441420	4	Lung laceration: with blood loss >20% by volume
441424	5	Lung laceration: with parenchymal laceration with massive air leak
441426	5	Lung laceration: with systemic air embolus
441436	4	Lung laceration: with blood loss >20% by volume
441440	5	Lung laceration: unilateral: with parenchymal laceration with massive air leak
441442	5	Lung laceration: unilateral: with systemic air embolus
441456	5	Lung laceration: bilateral: with blood loss >20% by volume
441460	5	Lung laceration: bilateral: with parenchymal laceration with massive air leak
441462	5	Lung laceration: bilateral: with systemic air embolus
441604	3	Pericardium: injury with tamponade without heart injury
441606	5	Pericardium: herniation of heart
442208	4	Thoracic cavity injury: with blood loss >20% by volume
442212	5	Thoracic cavity injury: with systemic air embolism
442699	3	Trachea and Main Stem Bronchus NFS
442602	3	Trachea and Main Stem Bronchus: contusion (hematoma)
442604	3	Trachea and Main Stem Bronchus: laceration NFS
442606	3	Trachea and Main Stem Bronchus: laceration: no perforation; partial thickness
442608	4	Trachea and Main Stem Bronchus: laceration: perforation; full thickness but not complete transection
442610	5	Trachea and Main Stem Bronchus: laceration: complex; avulsion; rupture; transection
442612	4	Trachea and Main Stem Bronchus: fracture NFS
442614	4	Trachea and Main Stem Bronchus: simple
442616	5	Trachea and Main Stem Bronchus: major with laryngeal-tracheal separation

NFS, not further specified

© 2020 Bowman JA et al. *JAMA Network Open.*

eTable 2. Thoracic AIS Predot Codes (2008 Update of the 2005 Version) Used for Exclusion Criteria in the 2016 NTDB Data Set

AIS "predot" code	AIS Score	Description
413000	6	Crush injury
415000	4	Open ("sucking") chest wound
416006	3	Penetrating injury with blood loss >20% by volume
410606	3	Skin/subcutaneous/muscle: laceration, blood loss >20% b volume
410806	3	Skin/subcutaneous/muscle: avulsion, blood loss >20% by volume
420299	4	Aorta, thoracic, NFS
420202	4	Aorta, thoracic: intimal tear, no disruption
420204	5	Aorta, thoracic: intimal tear, no disruption, with aortic valve involvement
420206	4	Aorta, thoracic: laceration; perforation; puncture, NFS
420208	4	Aorta, thoracic: laceration; perforation; puncture: minor; superficial; incomplete circumferential involvement; blood loss ≤20% by volume
420210	5	Aorta, thoracic: laceration; perforation; puncture: major; rupture; transection; segmental loss; blood loss >20% by volume
420212	5	Aorta, thoracic: laceration; perforation; puncture: major; with aortic root or valve involvement
420216	5	Aorta, thoracic: laceration; perforation; puncture: major, with hemorrhage confined to mediastinum
420218	6	Aorta, thoracic: laceration; perforation; puncture: major, with hemorrhage not confined to mediastinum
420499	3	Brachiocephalic (innominate) artery NFS
420402	3	Brachiocephalic (innominate) artery: intimal tear, no disruption
420404	3	Brachiocephalic (innominate) artery: laceration; perforation; puncture, NFS
420406	3	Brachiocephalic (innominate) artery: laceration; perforation; puncture: minor; superficial; incomplete circumferential involvement; blood loss ≤20% by volume
420408	4	Brachiocephalic (innominate) artery: laceration; perforation; puncture: major; rupture; transection; segmental loss; blood loss >20% by volume
420800	5	Coronary artery laceration or thrombosis to left main, right main or left anterior descending artery; coronary sinus
421099	3	Pulmonary artery NFS
421002	3	Pulmonary artery: intimal tear, no disruption
421004	3	Pulmonary artery: laceration; perforation; puncture, NFS
421006	3	Pulmonary artery: laceration; perforation; puncture: minor; superficial; incomplete circumferential involvement; blood loss ≤20% by volume
421008	5	Pulmonary artery: laceration; perforation; puncture: major; rupture; transection; segmental loss; blood loss >20% by volume
421009	6	Pulmonary artery: laceration; perforation; puncture: major; bilateral
421499	3	Subclavian artery NFS
421402	3	Subclavian artery: intimal tear, no disruption
421404	3	Subclavian artery: laceration; perforation; puncture, NFS
421406	3	Subclavian artery: laceration; perforation; puncture: minor; superficial; incomplete circumferential involvement; blood loss ≤20% by volume

© 2020 Bowman JA et al. *JAMA Network Open.*

eTable 2. Thoracic AIS Predot Codes (2008 Update of the 2005 Version) Used for Exclusion Criteria in the 2016 NTDB Data Set (continued).

AIS "predot" code	AIS Score	Description
421408	4	Subclavian artery: laceration; perforation; puncture: major; rupture; transection; segmental blood loss >20% by volume
422008	3	Other named arteries: laceration; perforation; puncture: major; rupture; transection; segmental loss; blood loss >20% by volume
420699	3	Brachiocephalic (innominate) vein NFS
420602	3	Brachiocephalic (innominate) vein: laceration; perforation; puncture, NFS
420604	3	Brachiocephalic (innominate) vein: laceration; perforation; puncture: minor; superficial; incomplete circumferential involvement; blood loss ≤20% by volume
420606	4	Brachiocephalic (innominate) vein: laceration; perforation; puncture: major; rupture; transection; segmental loss; blood loss >20% by volume
420608	5	Brachiocephalic (innominate) vein: laceration; perforation; puncture: major, with air embolus right side
421299	3	Pulmonary vein NFS
421202	3	Pulmonary vein: laceration; perforation; puncture, NFS
421204	3	Pulmonary vein: laceration; perforation; puncture: minor; superficial; incomplete circumferential involvement; blood loss ≤20% by volume
421206	5	Pulmonary vein: laceration; perforation; puncture: major; rupture; transection; segmental loss; blood loss >20% by volume
421207	6	Pulmonary vein: laceration; perforation; puncture: major; bilateral
421699	3	Subclavian vein NFS
421602	3	Subclavian vein: laceration; perforation; puncture: NFS
421604	3	Subclavian vein: laceration; perforation; puncture: minor; superficial; incomplete circumferential involvement; blood loss ≤20% by volume
421606	4	Subclavian vein: laceration; perforation; puncture: major; rupture; transection; segmental loss; blood loss >20% by volume
421899	3	Vena Cava, superior and thoracic portion of inferior, NFS
421802	3	Vena Cava, superior and thoracic portion of inferior: laceration; perforation; puncture, NFS
421804	3	Vena Cava, superior and thoracic portion of inferior: laceration; perforation; puncture: minor; superficial; incomplete circumferential involvement; blood loss ≤20% by volume
421806	4	Vena Cava, superior and thoracic portion of inferior: laceration perforation; puncture: major; rupture; transection; segmental loss; blood loss >20% by volume
421808	5	Vena Cava, superior and thoracic portion of inferior: laceration perforation; puncture: major, with air embolus right side
422206	3	Other named veins NFS: laceration; perforation; puncture: major; rupture; transection; segmental loss; blood loss >20% by volume
440199	3	Bronchus, main stem, NFS
440102	3	Bronchus, main stem: contusion; hematoma
440104	3	Bronchus, main stem: laceration; tear, NFS
440106	3	Bronchus, main stem: laceration; tear: no perforation; partial thickness
440108	4	Bronchus, main stem: laceration; tear: perforation; full thickness; "fracture"
440110	5	Bronchus, main stem: laceration; tear: complex; avulsion; rupture; transection; with separation

eTable 2. Thoracic AIS Predot Codes (2008 Update of the 2005 Version) Used for Exclusion Criteria in the 2016 NTDB Data Set (continued).

AIS "predot" code	AIS Score	Description
440208	3	Bronchus distal to main stem: laceration; tear: perforation; full thickness; "fracture"
440210	4	Bronchus distal to main stem: laceration; tear: complex; avulsion; rupture; transection; with separation
440606	3	Diaphragm: laceration ≤10 cm
440608	4	Diaphragm: laceration >10 cm; with significant tissue loss
440610	4	Diaphragm: rupture with herniation
440805	3	Esophagus injury in thorax: ingestion injury NFS
440807	3	Esophagus injury in thorax: ingestion injury: partial-thickness necrosis
440809	4	Esophagus injury in thorax: ingestion injury: full-thickness necrosis
440804	3	Esophagus: laceration; tear NFS
440806	3	Esophagus: laceration; tear: no perforation; partial thickness; ≤50% circumference
440808	4	Esophagus: laceration; tear: perforation; full thickness; >50% circumference
440810	5	Esophagus: laceration; tear: avulsion; rupture; transection; massive destruction
441006	4	Heart (Myocardium): contusion: major
441008	3	Heart (Myocardium): laceration NFS
441010	3	Heart (Myocardium): laceration: no perforation, no chamber involvement
441012	5	Heart (Myocardium): laceration: perforation, ventricular or atrial, with or without tamponade
441013	5	Heart (Myocardium): laceration: perforation, with atrial rupture
441014	6	Heart (Myocardium): laceration: perforation, with ventricular rupture
441016	6	Heart (Myocardium): laceration: perforation: multiple lacerations; >50% tissue loss of a chamber
441018	6	Heart (Myocardium): avulsion
440400	5	Intracardiac chordae tendineae laceration; rupture
441300	5	Intracardiac septum laceration; rupture
441200	5	Intracardiac valve laceration; rupture
441499	3	Lung NFS
441426	5	Lung: blast injury: severe; bilateral with air embolus
419202	3	Lung: inhalation injury: minor or patchy areas of erythema, bronchorrhea, carbonaceous deposits in proximal or distal bronchi
419204	4	Lung: inhalation injury: moderate degree of erythema, carbonaceous deposits, bronchorrhea with or without compromise of the bronchi
419206	5	Lung: inhalation injury: severe inflammation with friability, copious carbonaceous deposits, bronchorrhea, bronchial obstruction, hypoxemia
419208	6	Lung: inhalation injury: evidence of mucosal sloughing, necrosis, endoluminal obliteration
441603	3	Pericardium: hemopericardium NFS
441604	3	Pericardium: hemopericardium: without cardiac tamponade or heart injury
441605	4	Pericardium: hemopericardium: with cardiac tamponade but without heart injury

© 2020 Bowman JA et al. *JAMA Network Open.*

eTable 2. Thoracic AIS Predot Codes (2008 Update of the 2005 Version) Used for Exclusion Criteria in the 2016 NTDB Data Set (continued).

AIS "predot" code	AIS Score	Description
441606	5	Pericardium: hemopericardium: herniation of heart through pericardium
442201	4	Thoracic injury: hemothorax: major; >1000cc blood loss on at least one side
442206	4	Thoracic injury: hemopneumothorax: major; >1000cc blood loss on at least one side
442207	5	Thoracic injury: air embolus
442210	3	Thoracic injury: pneumomediastinum: with cardiac tamponade
442699	3	Trachea injury in thorax, NFS
442602	3	Trachea injury in thorax: contusion; hematoma
442604	3	Trachea injury in thorax: laceration, NFS
442606	3	Trachea injury in thorax: laceration: no perforation; partial thickness
442608	4	Trachea injury in thorax: laceration: perforation; full thickness; "fracture"
442610	5	Trachea injury in thorax: laceration: avulsion; rupture; transection; massive destruction; crush; laryngeal-tracheal separation
451020	4	Thoracic wall: avulsion of chest wall tissues including rib cage
451021	4	Thoracic wall: avulsion: minor; ≤15% of chest wall including rib cage
451022	5	Thoracic wall: avulsion: major; >15% of chest wall including rib cage

NFS, not further specified

© 2020 Bowman JA et al. *JAMA Network Open.*

eTable 3. Baseline Physiologic Characteristics

	Hospital-level proportion of older patients with isolated rib fractures admitted to an ICU					
	Quartile 1	Quartile 2	Quartile 3	Quartile 4		Standardized difference between Quartiles 1 and 4
	0-7.3%	7.4-16.5%	16.6-32.0%	32.1-91.9%		
Patient characteristic	n = 5,336	n = 5,548	n = 5,597	n = 7,470	*P* value	
Systolic blood pressure at presentation (mmHg), n (%)					0.65	-0.02
<90	54 (1.0)	54 (1.0)	65 (1.2)	70 (0.9)		
≥90	5,206 (97.6)	5,411 (97.5)	5,438 (97.2)	7,271 (97.3)		
Not recorded	76 (1.4)	83 (1.5)	94 (1.7)	129 (1.7)		
Heart rate at presentation (beats per minute), n (%)					<0.001	-0.08
<50	34 (0.6)	38 (0.7)	34 (0.6)	57 (0.8)		
50-99	4,584 (85.9)	4,632 (83.5)	4,706 (84.1)	6,203 (83.0)		
≥100	652 (12.2)	758 (13.7)	772 (13.8)	1,079 (14.4)		
Not recorded	66 (1.2)	120 (2.2)	85 (1.5)	131 (1.8)		
Respiratory rate at presentation (breaths per minute), n (%)					0.56	-0.01
<12	41 (0.8)	36 (0.6)	44 (0.8)	47 (0.6)		
12-24	4,917 (92.2)	5,130 (92.5)	5,144 (91.9)	6,882 (92.1)		
>24	266 (5.0)	276 (5.0)	295 (5.3)	372 (5.0)		
Not recorded	112 (2.1)	106 (1.9)	114 (2.0)	169 (2.3)		
Oxygen saturation at presentation (%), n (%)					<0.001	-0.09
<90	293 (5.5)	265 (4.8)	244 (4.4)	261 (3.5)		
90-95	1,830 (34.3)	1,783 (32.1)	1,786 (31.9)	2,355 (31.5)		
96-100	3,076 (57.6)	3,337 (60.2)	3,207 (57.3)	4,370 (58.5)		
Not recorded	137 (2.6)	163 (2.9)	360 (6.4)	484 (6.5)		

ICU, intensive care unit

© 2020 Bowman JA et al. *JAMA Network Open.*

eTable 4. Strength of Instrumental Variables Based on Hospital ICU Utilization in Predicting ICU Admission for Individual Patients

Outcome	Unadjusted			Adjusted		
	Risk Ratio	95% CI	Semi-partial R^2	Risk Ratio	95% CI	Semi-partial R^2
**Composite**						
IV_1: Median groups	0.17	0.16-0.18	0.088	0.21	0.20-0.22	0.1019
IV_2: Tertile groups	0.12	0.11-0.13	0.134	0.15	0.14-0.16	0.0914
IV_3: Quartile groups	0.09	0.08-0.10	0.165	0.13	0.12-0.14	0.0905
**Death**						
IV_1: Median groups	0.17	0.16-0.18	0.088	0.21	0.20-0.22	0.1020
IV_2: Tertile groups	0.12	0.11-0.13	0.134	0.15	0.14-0.16	0.0910
IV_3: Quartile groups	0.09	0.08-0.10	0.165	0.13	0.12-0.14	0.0900
**Pneumonia**						
IV_1: Median groups	0.17	0.16-0.18	0.088	0.21	0.10-0.22	0.1019
IV_2: Tertile groups	0.12	0.11-0.13	0.134	0.15	0.14-0.16	0.0914
IV_3: Quartile groups	0.09	0.08-0.10	0.165	0.13	0.12-0.14	0.0904
**Unplanned intubation**						
IV_1: Median groups	0.17	0.16-0.18	0.088	0.21	0.20-0.22	0.1019
IV_2: Tertile groups	0.12	0.11-0.13	0.134	0.15	0.14-0.16	0.0914
IV_3: Quartile groups	0.09	0.08-0.10	0.165	0.13	0.12-0.14	0.0005
**ARDS**						
IV_1: Median groups	0.17	0.16-0.18	0.088	0.21	0.19-0.22	0.1019
IV_2: Tertile groups	0.12	0.11-0.13	0.134	0.15	0.14-0.16	0.0914
IV_3: Quartile groups	0.09	0.08-0.10	0.165	0.13	0.12-0.14	0.0905
**Unplanned ICU transfer**						
IV_1: Median groups	0.17	0.16-0.18	0.088	0.21	0.20-0.22	0.1019
IV_2: Tertile groups	0.12	0.11-0.13	0.134	0.15	0.14-0.16	0.0914
IV_3: Quartile groups	0.09	0.08-0.10	0.165	0.13	0.12-0.14	0.0904

All comparisons represent the highest quantile relative to the lowest; IV, instrumental variable.

© 2020 Bowman JA et al. *JAMA Network Open.*

eTable 5. Covariate Balance by Exposure and Instrument Status

Covariate	Standardized Difference[a]		
	IV_1: Median groups	IV_2: Tertile groups	IV_3: Quartile groups
Age in categories	0.0445	0.0633	0.121
Gender	0.0263	0.0209	0.0247
Injury location (type of place where injury occurred)	0.1605	0.2384	0.2077
Injury type	0.0483	0.1682	0
Injury intent	0.0483	0.1495	0.1495
Injury mechanism	0.251	0.3205	0.2596
Injury mechanism in categories	0.1428	0.1927	0.2039
Type of transportation from scene of injury to hospital	0.1437	0.2575	0.2133
Patient transferred from another hospital	0.1891	0.266	0.3466
Payer in categories	0.2929	0.2182	0.2543
Comorbidity: chronic obstructive pulmonary disease	-0.0273	-0.0235	0.0018
Comorbidity: smoking	0.0026	0.0222	0.0323
Comorbidity: functional dependency	0.01	0.0244	0.0501
Comorbidity: other	-0.0643	-0.0892	-0.0842
Comorbidity: alcohol use disorder	-0.0067	-0.024	0.0149
Comorbidity: bleeding disorder	-0.0208	-0.0182	-0.0062
Comorbidity: chemotherapy	-0.0017	0.0076	0.0053
Comorbidity: congenital anomaly	0.0361	0.0236	0.0263
Comorbidity: cerebrovascular accident (stroke)	0.0058	0.001	0.0203
Comorbidity: diabetes mellitus	0.0113	0.0214	0.0135
Comorbidity: disseminated cancer	-0.0292	-0.0387	-0.0489
Comorbidity: limitation on care (e.g., advanced directive)	0.0024	-0.0179	-0.0082
Comorbidity: angina	0.0125	0.0111	0.0154
Comorbidity: myocardial infarction	-0.0138	-0.0339	-0.0266
Comorbidity: peripheral vascular disease	-0.0054	0.0085	0.0229
Comorbidity: hypertension	0.0031	0.0443	0.0788
Comorbidity: prematurity	0.0125	0.0146	0.0166
Comorbidity: steroid use	0.0133	0.019	0.0189
Comorbidity: cirrhosis	0.029	0.0562	0.0691
Comorbidity: dementia	-0.0297	-0.0365	-0.0014
Comorbidity: drug use disorder	-0.0059	-0.0169	-0.0246
Comorbidity: attention deficit hyperactivity disorder	0.0032	0.0027	0.0186
Alcohol use in categories	0.1278	0.2437	0.2106
Sedation/paralytic medication administered	0.0603	0.0852	0.116
Systolic blood pressure in categories	0.0823	0	0.0823

© 2020 Bowman JA et al. *JAMA Network Open.*

eTable 5. Covariate Balance by Exposure and Instrument Status (continued).

Covariate	Standardized Difference[a]		
	IV_1: Median groups	IV_2: Tertile groups	IV_3: Quartile groups
Heart rate in categories	0.0889	0.0889	0.1041
Respiratory rate in categories	0.0713	0.0483	0
Oxygen saturation in categories	0.2965	0.3246	0.2978
Supplemental oxygen use in categories	0.2487	0.2901	0.2741
Glasgow Coma Scale total score in categories	0	0	0.0406
Glasgow Coma Scale total score	0.044	0.044	0.058
Injury Severity Score in categories	0.0443	0.1697	0.1768
Abbreviated Injury Scale score: head	-0.023	-0.0401	-0.0335
Abbreviated Injury Scale score: face	0.0336	0.0242	0.0302
Abbreviated Injury Scale score: neck	0.003	0.0141	0.0226
Abbreviated Injury Scale score: thorax	0.244	.	.
Abbreviated Injury Scale score: abdomen	-0.0056	-0.0024	0.0102
Abbreviated Injury Scale score: spine	-0.0288	-0.0436	-0.0305
Abbreviated Injury Scale score: upper extremities	-0.0007	-0.0142	-0.0026
Abbreviated Injury Scale score: lower extremities	0.0059	0.0177	0.0148
Abbreviated Injury Scale score: external	-0.0392	-0.0632	-0.0578
Hemopneumothorax by AIS codes	0.0126	0.0213	0.0292
Hemothorax by AIS codes	0.0071	0.005	-0.0032
Pneumothorax by AIS codes	-0.0059	-0.0224	-0.0183
Lung contusion by AIS codes	0.0385	0.0538	0.0571
Lung laceration by AIS codes	0.0134	0.0308	0.0284
Pneumomediastinum by AIS codes	0.0191	0.0171	0.0217
Hemomediastinum by AIS codes	0.0077	0.0227	0.0286
Flail chest by AIS codes	0.0485	0.0598	0.0727
Sternal fracture by AIS codes	0.0624	0.0782	0.0893
Hemopneumothorax by ICD codes	0.0207	0.0343	0.0437
Hemothorax by ICD codes	0.0399	0.0691	0.0808
Pneumothorax by ICD codes	0.0206	0.0257	0.0336
Lung contusion by ICD codes	0.0478	0.0651	0.0699
Lung laceration by ICD codes	0.018	0.0369	0.0451
Open rib fracture by ICD codes	0.0136	0.0274	0.0218
Flail chest by ICD codes	0.0505	0.0686	0.0811
Sternal fracture by ICD codes	0.0648	0.083	0.0945
Clavicle fracture by ICD codes	0.0691	0.0702	0.0725
Scapula fracture by ICD codes	0.0527	0.0468	0.0644
Hemopneumothorax by either AIS or ICD codes	0.0275	0.0358	0.0446

© 2020 Bowman JA et al. *JAMA Network Open.*

eTable 5. Covariate Balance by Exposure and Instrument Status (continued).

Covariate	Standardized Difference[a]		
	IV_1: Median groups	IV_2: Tertile groups	IV_3: Quartile groups
Hemothorax by either AIS or ICD codes	0.0385	0.0651	0.0767
Pneumothorax by either AIS or ICD codes	0.025	0.0259	0.0335
Lung contusion by either AIS or ICD codes	0.0487	0.0653	0.0697
Lung laceration by either AIS or ICD codes	0.0165	0.0326	0.0343
Flail chest by either AIS or ICD codes	0.0533	0.0645	0.0799
Sternal fracture by either AIS or ICD codes	0.0527	0.0468	0.0644
Number of fractured ribs by either AIS or ICD codes	0.1174	0.1182	0.1569
Chest tube placed	-0.0064	-0.0008	0.0127

IV, instrumental variable, AIS, Abbreviated Injury Scale; ICD, International Classification of Diseases
[a] <0.1 signifies covariate balance

eTable 6. Patient-Level and Hospital-Level Characteristics Incorporated Into the Propensity Scores

Level	Description	Variable format	Number of categories	Description of categories, if different than as defined or presented in the NTDB
patient	age in categories	categorical	5	65-69; 70-74; 75-79; 80-84; or 85-89 (years)
patient	gender	dichotomous	2	(as defined/presented in NTDB)
patient	injury location (type of place where injury occurred)	categorical	10	Home; farm; industry; recreation; street; public building; residential institution; other; unspecified; or location not known/not recorded
patient	injury type	categorical	5	(as defined/presented in NTDB)
patient	injury intent	categorical	6	(as defined/presented in NTDB)
patient	injury mechanism	categorical	24	(as defined/presented in NTDB)
patient	type of transportation from scene of injury to hospital	categorical	8	(as defined/presented in NTDB)
patient	patient transferred from another hospital	dichotomous	2	(as defined/presented in NTDB)
patient	payer in categories	categorical	9	(as defined/presented in NTDB)
patient	comorbidity: chronic obstructive pulmonary disease	dichotomous	2	(as defined/presented in NTDB)
patient	comorbidity: smoking	dichotomous	2	(as defined/presented in NTDB)
patient	comorbidity: functional dependency	dichotomous	2	(as defined/presented in NTDB)
patient	comorbidity: other	dichotomous	2	(as defined/presented in NTDB)
patient	comorbidity: alcohol use disorder	dichotomous	2	(as defined/presented in NTDB)
patient	comorbidity: bleeding disorder	dichotomous	2	(as defined/presented in NTDB)
patient	comorbidity: chemotherapy	dichotomous	2	(as defined/presented in NTDB)
patient	comorbidity: congenital anomaly	dichotomous	2	(as defined/presented in NTDB)
patient	comorbidity: cerebrovascular accident (stroke)	dichotomous	2	(as defined/presented in NTDB)
patient	comorbidity: diabetes mellitus	dichotomous	2	(as defined/presented in NTDB)
patient	comorbidity: disseminated cancer	dichotomous	2	(as defined/presented in NTDB)
patient	comorbidity: limitation on care (e.g., advanced directive)	dichotomous	2	(as defined/presented in NTDB)
patient	comorbidity: angina	dichotomous	2	(as defined/presented in NTDB)

© 2020 Bowman JA et al. *JAMA Network Open.*

eTable 6. Patient-Level and Hospital-Level Characteristics Incorporated Into the Propensity Scores (continued).

Level	Description	Variable format	Number of categories	Description of categories, if different than as defined or presented in the NTDB
patient	comorbidity: myocardial infarction	dichotomous	2	(as defined/presented in NTDB)
patient	comorbidity: peripheral vascular disease	dichotomous	2	(as defined/presented in NTDB)
patient	comorbidity: hypertension	dichotomous	2	(as defined/presented in NTDB)
patient	comorbidity: prematurity	dichotomous	2	(as defined/presented in NTDB)
patient	comorbidity: steroid use	dichotomous	2	(as defined/presented in NTDB)
patient	comorbidity: cirrhosis	dichotomous	2	(as defined/presented in NTDB)
patient	comorbidity: dementia	dichotomous	2	(as defined/presented in NTDB)
patient	comorbidity: drug use disorder	dichotomous	2	(as defined/presented in NTDB)
patient	comorbidity: attention deficit hyperactivity disorder	dichotomous	2	(as defined/presented in NTDB)
patient	alcohol use in categories	categorical	6	(as defined/presented in NTDB)
patient	sedation/paralytic medication administered	dichotomous	2	(as defined/presented in NTDB)
patient	systolic blood pressure in categories	categorical	3	≥90, <90, or not known/recorded (mmHg)
patient	heart rate in categories	categorical	4	<50, 50-99, ≥100, or not known/recorded (beats/minute)
patient	respiratory rate in categories	categorical	8	0-5, 6-9, 10-11, 12-24, 25-34, 35-49, ≥50, or not known/recorded (respirations/minute)
patient	oxygen saturation in categories	categorical	5	<80, 80-89, 90-95, 96-100, or not known/recorded (%)
patient	supplemental oxygen use in categories	categorical	4	(as defined/presented in NTDB)
patient	Glasgow Coma Scale total score	categorical	9	(as defined/presented in NTDB)
patient	Injury Severity Score in categories	categorical	5	1-9, 10-16, 17-24, 25+, or not described
patient	Abbreviated Injury Scale score: head	dichotomous	2	(as defined/presented in NTDB)
patient	Abbreviated Injury Scale score: face	dichotomous	2	(as defined/presented in NTDB)

© 2020 Bowman JA et al. *JAMA Network Open.*

eTable 6. Patient-Level and Hospital-Level Characteristics Incorporated Into the Propensity Scores (continued)s

Level	Description	Variable format	Number of categories	Description of categories, if different than as defined or presented in the NTDB
patient	Abbreviated Injury Scale score: neck	dichotomous	2	(as defined/presented in NTDB)
patient	Abbreviated Injury Scale score: thorax	categorical	6	(as defined/presented in NTDB)
patient	Abbreviated Injury Scale score: abdomen	dichotomous	2	(as defined/presented in NTDB)
patient	Abbreviated Injury Scale score: spine	dichotomous	2	(as defined/presented in NTDB)
patient	Abbreviated Injury Scale score: upper extremities	dichotomous	2	(as defined/presented in NTDB)
patient	Abbreviated Injury Scale score: lower extremities	dichotomous	2	(as defined/presented in NTDB)
patient	Abbreviated Injury Scale score: external	dichotomous	2	(as defined/presented in NTDB)
patient	pneumomediastinum by AIS codes	dichotomous	2	(based on AIS codes, as presented in NTDB)
patient	hemomediastinum by AIS codes	dichotomous	2	(based on AIS codes, as presented in NTDB)
patient	open rib fracture by ICD codes	dichotomous	2	(based on ICD codes, as presented in NTDB)
patient	clavicle fracture by ICD codes	dichotomous	2	(based on ICD codes, as presented in NTDB)
patient	scapula fracture by ICD codes	dichotomous	2	(based on ICD codes, as presented in NTDB)
patient	hemopneumothorax by either AIS or ICD codes	dichotomous	2	(based on AIS and ICD codes, as presented in NTDB)
patient	hemothorax by either AIS or ICD codes	dichotomous	2	(based on AIS and ICD codes, as presented in NTDB)
patient	pneumothorax by either AIS or ICD codes	dichotomous	2	(based on AIS and ICD codes, as presented in NTDB)
patient	lung contusion by either AIS or ICD codes	dichotomous	2	(based on AIS and ICD codes, as presented in NTDB)
patient	lung laceration by either AIS or ICD codes	dichotomous	2	(based on AIS and ICD codes, as presented in NTDB)
patient	flail chest by either AIS or ICD codes	dichotomous	2	(based on AIS and ICD codes, as presented in NTDB)
patient	sternal fracture by either AIS or ICD codes	dichotomous	2	(based on AIS and ICD codes, as presented in NTDB)

© 2020 Bowman JA et al. *JAMA Network Open.*

eTable 6. Patient-Level and Hospital-Level Characteristics Incorporated Into the Propensity Scores (continued)

Level	Description	Variable format	Number of categories	Description of categories, if different than as defined or presented in the NTDB
patient	number of fractured ribs by either AIS or ICD codes	categorical	10	1; 2; 3; 4; 5; 6; 7; 8+; multiple; or unspecified number of fractured ribs
patient	chest tube placed	dichotomous	2	(based on ICD codes)
hospital	trauma center level	categorical	5	I; II; III; IV; or not applicable (as determined by American College of Surgeons level or state level designation, with American College of Surgeons level trumping state level)
hospital	teaching status of hospital	categorical	3	(as defined/presented in NTDB)
hospital	region of country in categories	categorical	5	(as defined/presented in NTDB)
hospital	number of adult inpatient beds, in categories	categorical	6	(as defined/presented in NTDB)
hospital	number of ICU beds, in categories	categorical	7	(as defined/presented in NTDB)
hospital	number of attending trauma surgeons, in categories	categorical	5	(as defined/presented in NTDB)
hospital	volume of aged patients with isolated rib fractures, in categories	categorical	5	10-19; 20-39; 40-59; 60-79; or 80+ eligible hospitalizations/year
hospital	presence of stepdown unit	dichotomous	2	(as determined empirically from the data)
hospital	volume of aged injured patients, in categories	categorical	6	0-<10, 10-<20, 20-<30, 30-<40, 40-<50, 50-100 (%)
hospital	volume of patients with blunt injury, in categories	categorical	6	0-<75, 75-<80, 80-<85, 85-<90, 90-<95, 95-100 (%)

NTDB, National Trauma Data Bank; AIS, Abbreviated Injury Scale; ICD, International Classification of Diseases; ICU, intensive care unit

© 2020 Bowman JA et al. *JAMA Network Open.*

eFigure. Rank Order Plot of Hospital-Level ICU Use

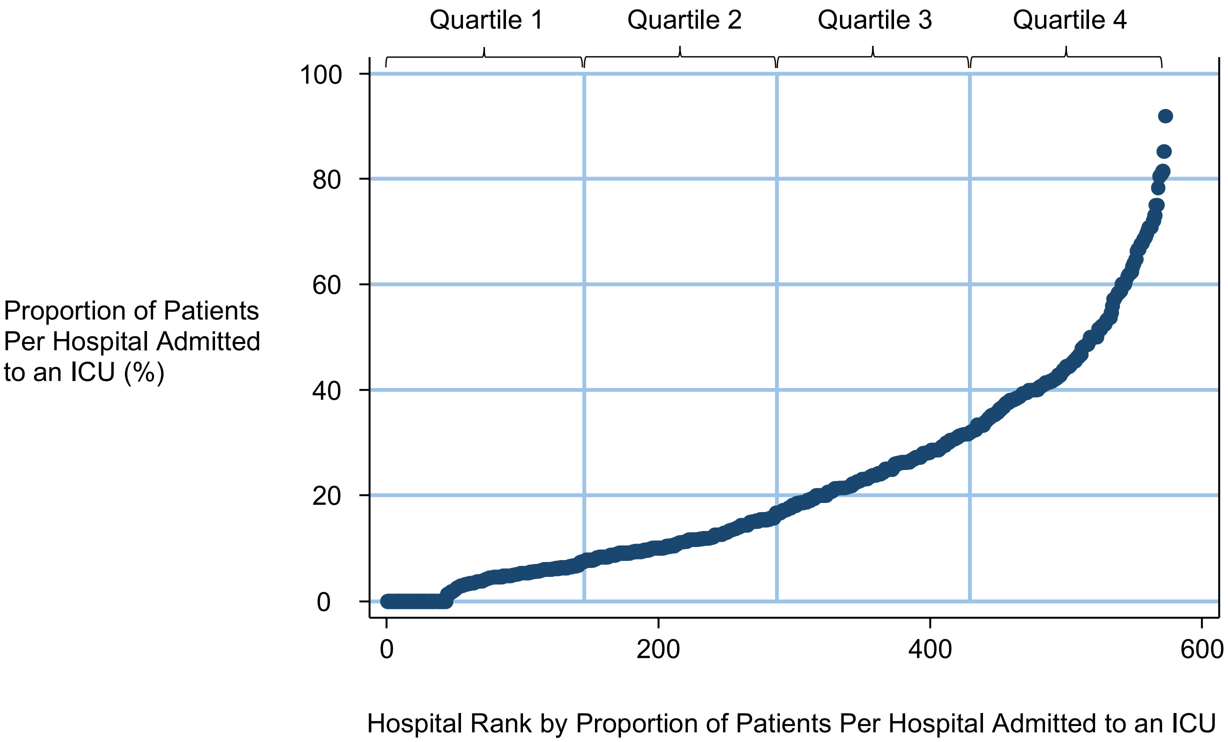

Each plotted point represents a hospital included in the study. Brackets represent the quartiles used for the analysis: quartile 1 includes hospitals with 0.0-7.3% of older patients with isolated rib fractures admitted to an ICU, quartile 2 includes hospitals with 7.4-16.5% admitted to an ICU, quartile 3 includes hospitals with 16.6-32.0% admitted to an ICU, and quartile 4 includes hospitals with 32.1-91.9% admitted to an ICU.

© 2020 Bowman JA et al. *JAMA Network Open.*
